# Modulatory Actions of the Glycine Receptor β Subunit on the Positive Allosteric Modulation of Ethanol in α2 Containing Receptors

**DOI:** 10.3389/fnmol.2021.763868

**Published:** 2021-11-18

**Authors:** Braulio Muñoz, Trinidad Mariqueo, Pablo Murath, Christian Peters, Gonzalo E. Yevenes, Gustavo Moraga-Cid, Robert W. Peoples, Luis G. Aguayo

**Affiliations:** ^1^Laboratory of Neurophysiology, Department of Physiology, Universidad de Concepción, Concepción, Chile; ^2^Laboratory of Neuropharmacology, Department of Physiology, Universidad de Concepción, Concepción, Chile; ^3^Department of Physiology, Universidad de Concepción, Concepción, Chile; ^4^Department of Biomedical Sciences, Marquette University, Milwaukee, WI, United States

**Keywords:** receptor pharmacology, glycine receptor, ethanol, allosteric modulation, G-protein

## Abstract

Alpha1-containing glycine receptors (GlyRs) are major mediators of synaptic inhibition in the spinal cord and brain stem. Recent studies reported the presence of α2-containing GlyRs in other brain regions, such as nucleus accumbens and cerebral cortex. GlyR activation decreases neuronal excitability associated with sensorial information, motor control, and respiratory functions; all of which are significantly altered during ethanol intoxication. We evaluated the role of β GlyR subunits and of two basic amino acid residues, K389 and R390, located in the large intracellular loop (IL) of the α2 GlyR subunit, which are important for binding and functional modulation by Gβγ, the dimer of the trimeric G protein conformation, using HEK-293 transfected cells combined with patch clamp electrophysiology. We demonstrate a new modulatory role of the β subunit on ethanol sensitivity of α2 subunits. Specifically, we found a differential allosteric modulation in homomeric α2 GlyRs compared with the α2β heteromeric conformation. Indeed, while α2 was insensitive, α2β GlyRs were substantially potentiated by ethanol, GTP-γ-S, propofol, Zn^2+^ and trichloroethanol. Furthermore, a Gβγ scavenger (ct-GRK2) selectively attenuated the effects of ethanol on recombinant α2β GlyRs. Mutations in an α2 GlyR co-expressed with the β subunit (α2AAβ) specifically blocked ethanol sensitivity, but not propofol potentiation. These results show a selective mechanism for low ethanol concentration effects on homomeric and heteromeric conformations of α2 GlyRs and provide a new mechanism for ethanol pharmacology, which is relevant to upper brain regions where α2 GlyRs are abundantly expressed.

## Introduction

Alcohol use disorder and alcoholism are major health problems affecting millions of people worldwide and causing great medical and economic burdens. Ethanol is a CNS depressant drug, and at intoxicating concentrations, it disrupts most brain functions including executive planning, awareness, muscle control, and memory (Spanagel, 2009). Inhibitory glycine receptors (GlyRs) play a central role controlling spinal and brain stem excitability (Legendre, 2001; Harvey et al., 2004; Lynch, 2004), and it is widely accepted that pharmacologically relevant concentrations of ethanol positively modulate α1 containing GlyRs (Aguayo and Pancetti, 1994; Eggers et al., 2000; Sebe et al., 2003).

More recently, it was found that GlyRs in the nucleus accumbens (nAc) might be implicated in ethanol intake and seeking behaviors (Molander and Soderpalm, 2005; Adermark et al., 2011; Li et al., 2012; Munoz et al., 2020). Accumbal neurons appear to express a mixed population of α1 and α2 subunits, however, it is largely unknown if they are equally sensitive to ethanol. Up to now, most studies that have examined the effects of ethanol on recombinant GlyRs have used homomeric conformations of α1 or α2 expressed in HEK 293 cells or oocytes (Crawford et al., 2008; Yevenes et al., 2010; McCracken et al., 2013). The studies showed that the α2 subunit was less sensitive to ethanol than α1 homomeric subunits (Yevenes et al., 2010). Furthermore, these studies indicated that although the intracellular loop (IL) molecular requirements are present in the α2 subunit, the channel is not a target for positive allosteric modulation by ethanol (Yevenes et al., 2010).

From the available results, we have been able to initiate our understanding on how ethanol sensitivity of the different GlyRs subunits relate to behaviors. For instance, Knock In (KI) mice with mutations in the IL of the α1 and α2 subunits showed a 30% shorter duration of loss of righting reflex (LORR) to ethanol compared to WT mice (Aguayo et al., 2014; Gallegos et al., 2021). In addition, KI mice showed higher intake of ethanol upon first exposure and greater conditioned place preference to ethanol (Munoz et al., 2020).

The present study shows that the β subunit is a key molecular component that affects ethanol sensitivity since co-expression of α2 with β subunits increased the sensitivity to low ethanol concentrations opening a new mechanistic alternative to alter the effect of ethanol in higher brain regions that express α2β GlyRs (Avila et al., 2013a, b). Thus, our study provides a new role for α2 and β subunits and reveals a previously undefined aspect of GlyRs pharmacology.

## Materials and Methods

### Cell Culture and Transfection

Human embryonic kidney (HEK) 293 cells (CRL-1573; American Type Culture Collection, Manassas, VA, United States) were cultured using standard methods. The cells were transfected using the calcium phosphate technique with 2 μg of cDNA plasmids per 35 mm dish encoding GlyR α subunits and 1 μg of EGFP. To favor the formation of heteromeric GlyRs, we transfected 1 μg of α subunits/EGFP plasmids plus 4 μg of β subunit cDNA (Yevenes et al., 2010). For the Gβγ sequester study, 1 μg of ct-GRK2 was co-transfected with GlyR α2 and β subunits. All recordings were made 18–24 h after transfection. The cDNA encoding the GlyRs has been described previously (Yevenes and Zeilhofer, 2011b). Residues in GlyR α2 (K389A and R390A) were replaced by alanine (α2AA) using the QuickChange site-directed mutagenesis kit (Agilent Technologies). Proper sequences of all constructs were confirmed by full-length sequencing.

### For Single Channel Recordings

HEK-293 cells were cultured to 70–95% confluence in minimum essential medium (MEM) containing 10% heat-inactivated donor horse serum, Earle’s salts, non-essential amino acids, sodium pyruvate, and GlutaMAX (Thermo Fisher Scientific) at 37°C with 5% CO_2_. Cells were plated in 35-mm dishes coated with poly-D-lysine and fibronectin and transfected with cDNA for the glycine receptors α1, α2, or α2β subunits and green fluorescent protein (Addgene) using a calcium phosphate transfection kit (Thermo Fisher Scientific). The cDNA ratios were 1:5 for α1: GFP, 1:10 for α2: GFP, and 1:10:2.5 for α2: β: GFP. The higher β plasmid ratio ensured heterometric GlyR formation for recordings that were done within 48 h following transfection. In our single channel recordings, conductance measurements supported the presence of heteromeric α2β receptors with higher conductance (100 pS). In addition, previous studies showed that incorporation of β to α2 subunits reduced the effects of picrotoxinoides (Fuentealba et al., 2011).

### Electrophysiology

Glycine-evoked currents were recorded from transfected HEK 293 cells in the whole-cell voltage-clamp configuration at room temperature (20–24°C) at a holding potential of −60 mV (Yevenes et al., 2010). Patch electrodes were pulled from borosilicate glass and were filled with (in mM): 120 CsCl, 10 BAPTA, 10 HEPES (pH 7.4), 4 MgCl_2_, 0.5 GTP, and 2 ATP. The external solution contained (in mM): 140 NaCl, 5.4 KCl, 2.0 CaCl_2_, 1.0 MgCl_2_, 10 HEPES (pH 7.4), and 10 glucose. Whole-cell recordings were performed with an Axoclamp 200B amplifier (Molecular Devices, United States) and acquired using Clampex 10.1 software. Data analysis was performed off-line using Clampfit 10.1 (Axon Instruments, Sunnyvale, CA, United States). Exogenous glycine-evoked currents were obtained using a stepper motor-driven rapid solution exchanger (Warner Instrument Corp). The percentages of rise and decay time were obtained from whole-cell current traces of 5 s of duration. The EC_10_ values for the recombinant and neuronal receptors were obtained experimentally after the successive application of increasing concentrations of glycine (1–1000 μM). The effects of ethanol or GTP-γ-S on the peak amplitude of the current were studied at an EC_10_ of glycine to compare the effects at equipotent concentrations. The concentration-response curve parameters (EC_50_ and Hill coefficients, n_h_) were obtained from the curve fits of normalized concentration–response data points to the equation I_agonist_ = I_max_ (agonist)^nh^ / [(agonist)^nh^ + (EC_50_)^nh^]. The mean maximal current (I_max_) indicated corresponds to the average maximal current elicited by saturating concentrations of the agonist. To study Gβγ activation, G proteins were activated with a non-hydrolyzable analog of GTP in the internal solution (GTP-γ-S, 0.5 mM, Sigma Aldrich).

The patch pipettes for single channel recordings had tip resistances of 7–15 MΩ and were manually fire polished in a microforge (Narishige, Japan). In some experiments they were coated with DuPont elastomer R6101 to reduce capacitive noise. Data was acquired using pClamp software and analyzed off-line with Clampfit 10.1 (Axon Instruments, Union City, CA, United States). Further details were previously published (Yevenes et al., 2008, 2010). Single-channel recording was performed at room temperature using an Axopatch 200B (Molecular Devices, Sunnyvale, CA, United States) amplifier and digitized with a 1322A Digidata (Axon Instruments, Union City, CA, United States). Data were acquired at 50 kHz and digitally low-pass filtered at 5 kHz. Outside-out patches were voltage-clamped at −60 mV and superfused in an external recording solution containing (in mM): 150 NaCl, 5 KCl, 0.2 CaCl_2_, 10 HEPES, 10 glucose, and 10 sucrose (pH 7.4). The intracellular recording solution contained (in mM): 140 CsCl, 10 EGTA, and 10 HEPES (pH 7.2). Solutions of glycine and ethanol were applied to patches using a stepper motor-driven solution exchange apparatus (Warner Instruments, Hamden, CT, United States) and 600 μm i.d. square glass tubing. Ethanol was alternately applied at 60 s intervals.

### Single Channel Analysis

Data records from single-channel recordings obtained from patches with one to three open levels were idealized using the segmentation K-means algorithm in the QUB software suite (Qin, 2004). The parameters analyzed were single channel conductance; MOT, mean open time; MST, mean shut time; Po, open probability. For overall mean open times and open probabilities (Po), values reported were obtained from these idealized records using Channelab (Synaptosoft). Data were obtained from 5 to 7 patches for each receptor subunit combination tested recorded for 2 min. Bursts were defined as openings or groups of openings that are likely to represent individual activations of the ion channel, and that were separated by shut times greater than a critical duration (τCrit). For burst analysis, shut time distributions were fitted with probability density functions using Channelab, and a τCrit value that minimized the total number of misclassified events was determined for each subunit combination tested. These values were 39 ms for α1 and 90 ms for α2β receptors. Groups of openings in idealized data records were then segmented into bursts using these values in QUB, excluding any segments of data with multi-level openings. The software programs Clampfit and Channelab were then used to fit probability density functions to distributions of burst durations as well as to intraburst open and shut events. Values for intraburst mean open and shut times and intraburst Po were obtained using Channelab.

### Reagents

Glycine (Sigma-Aldrich) was prepared in external solution at a stock concentration of 10 mM. Zinc chloride (Sigma-Aldrich) was prepared in H_2_O at a stock concentration of 10 mM. Ethanol (Merk-Millipore) and trichloroethanol was dissolved directly in the external solution. Propofol (Sigma-Aldrich) and isoflurane (Baxter) was dissolved in DMSO at a stock concentration of 100 mM and kept at −20°C.

### Sample Size

The target number of samples in each group for our electrophysiological experiments was determined based on findings reported in our previously published studies (Yevenes et al., 2006, 2008, 2010). Using these effect sizes and an α-level set at 0.05 and at 80% power, we determined that 5–7 electrophysiological recordings was an appropriate sample size.

### Replication

All sample sizes indicated in figures for electrophysiological experiments represent biological replicates.

### Data Analysis

All data was presented as mean ± standard error of means (SEM). The analyses were performed using two-tailed unpaired and two-tailed paired Student’s *t*-tests following an *F*-test to confirm similar variances. Non-normally distributed data were analyzed using two-tailed Welch’s tests for unpaired data. A two-way ANOVA test followed by Sidak’s multiple comparisons test was performed for Figures 2B,D, 5B. The value ^∗^*p* < 0.05 was considered statistically significant. All the statistical analysis and plots were performed with MicroCal Origin 8.0 (Northampton, MA, United States) and Prism 9.0 (GraphPad, La Jolla, CA, United States) software.

## Results

### The β Subunit Converts α2 Subunits to an “α1-Like” Glycine Receptor With Respect to Ethanol Sensitivity

**FIGURE 1:**
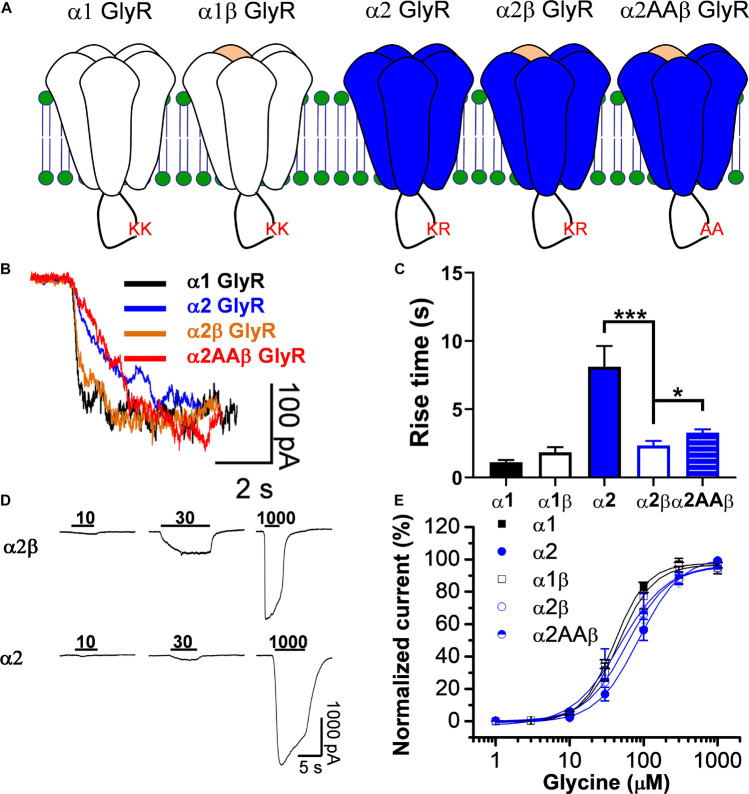
Co-expression of β subunits convert α2 GlyRs into an α1-like GlyRs. **(A)** Topology scheme of the glycine receptors used in the present study. Note that the intracellular loop in wild type α subunits present pairs of basic residues important for ethanol potentiation (KK or KR). **(B)** Representative traces of glycine-evoked currents, at EC_10_, showing the kinetic differences between homomeric and heteromeric GlyRs. **(C)** The bar graph shows the increase in rise time in α2 GlyRs, which is significantly reduced in heteromeric α2β GlyRs. **(D)** Representative glycine-evoked current traces showing the differences of glycine affinity between α2 and α2β GlyRs. **(E)** The graph shows the glycine concentration-response curve for all the GlyRs conformations. Values for EC_50_ can be found in Table 1. Data are mean ± SEM. *p < 0.05, ***p < 0.001. Unpaired Student’s *t* test.

Glycine receptors can be expressed in recombinant systems as homomeric or heteromeric complexes (4 α subunits:1 β subunit) (Yu et al., 2021; Zhu and Gouaux, 2021) and their expression can be monitored looking at changes on their properties such as time to activation and glycine affinity (Figure 1A). Figure 1B shows current traces activated by an EC_10_ concentration of glycine in homomeric and heteromeric GlyRs. In agreement to previous studies (Yevenes et al., 2010), this data shows that homomeric α1 GlyRs activate faster than the α2 GlyRs. Additionally, the co-expression of β with α2 resulted in an α2β complex that displayed a faster time course for activation, thus resulting in an α1-like phenotype (Figure 1C). As indicated in methods, we used a high β:α plasmid ratio to ensure the assembly of heteropentameric receptors (4 α subunits:1 β subunit). Analyses of concentration-response relationships show that the α2-homomeric GlyRs display a higher EC_50_ than the α1 GlyRs. In the α1 GlyRs, for example, the EC_50_ was 40 ± 1 μM (*n* = 10), while in α2 it was 86 ± 2 μM (*p* < 0.001, *n* = 10, Figure 1E and Table 1). In addition, co-expression of the β subunit decreased the EC_50_ in α2-containings GlyRs to 48 ± 8 μM (*n* = 10), without significant differences in α1β GlyRs (Figures 1D,E and Table 1). This decrease in EC_50_ in α2β GlyRs changes some properties of the α2-homomeric receptor complex, thus functionally it is an α1-like GlyRs. Because the WT subunits display two basic residues in the IL that are important for ethanol modulation (Yevenes et al., 2010; Munoz et al., 2020), we replaced the K389 and R390 residues in the WT α2 GlyR (α2AAβ) to test their role in the heteropentameric receptor (Gallegos et al., 2021). We found that the mutations did not cause large effects in the properties of the current (Figures 1C,E see legend for explanation of denoted residues in the IL).

**TABLE 1:** Properties of whole cell currents activated by several GlyRs conformations.

GlyR	EC_50_ (μM)	nH	Imax (pA)	Rise time (s)	10 mM ethanol potentiation (%)	100 mM ethanol potentiation (%)	GTP-γ-S potentiation (%)	Propofol potentiation (%)
α1	40 ± 1 (10)	1.96 ± 0.1	2111 ± 351	1.111 ± 0.1	11 ± 3 (16)	46 ± 4 (16) (**)	80 ± 10 (ref)	363 ± 33 (ref)
α1β	46 ± 2 (9)	1.85 ± 0.1	2355 ± 573	1.847 ± 0.3	9 ± 4 (10)	25 ± 5 (10)	ND	ND
α2	86 ± 2 (10) (***)	1.52 ± 0.1	2493 ± 359	8.116 ± 1.5	−4 ± 4 (12)	12 ± 4 (12)	−1 ± 7 (4)	30 ± 7 (6)
α2β	48 ± 8 (10) (***)	1.5 ± 0.1	3280 ± 471	2.339 ± 0.3 (***)	31 ± 7 (15) (***)	90 ± 20 (15) (**)	73 ± 11 (10) (**)	210 ± 50 (7)
α2AAβ	60 ± 1 (12)	1.5 ± 0.03	1676 ± 276	3.286 ± 0.2 (*)	12 ± 7 (8)	10 ± 6 (8)	−14 ± 12 (10)	306 ± 58 (8)

Values are given as mean ± SEM. Values were fitted to the equation I_glicine_ = I_max_ [glycine]^nH^/([glycine]^nH^+[EC_50_]^nH^) using Origin 8.0 software. The EC10 calculated for all subunits was used to measure rise time, decay time, ethanol and GTP-γ-S sensitivity experiment. “Ethanol potentiation” corresponds to the change between the control with glycine EC10 and presence of 10 and 100 mM ethanol. The “GTP-γ-S potentiation” corresponds to the change after 15 minutes of dialysis of the non-hydrolyzed analog, GTP-γ-S (200 μM). The “Propofol Potentiation” corresponds to the change between control with 30 μM Propofol. ND: Non determined *p < 0.05, **p < 0.01 and ***p <0.001, One way ANOVA (n) = number of cells.

**FIGURE 2:**
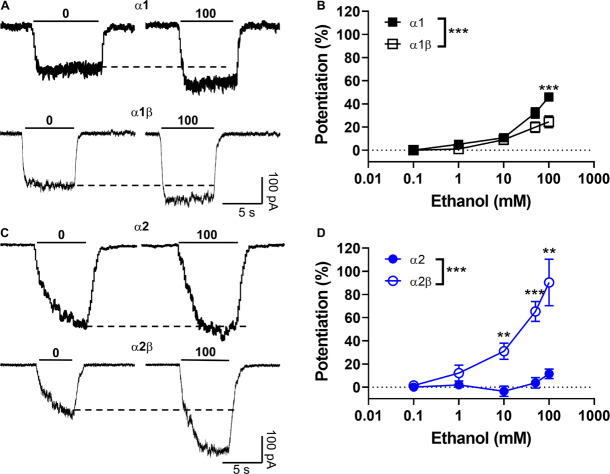
Modulatory effects of the beta subunit on the sensitivity of α2-expressing GlyRs to ethanol. **(A)** Representative current traces recorded in HEK cells expressing homomeric α1 and heteromeric α1β GlyRs and activated with an EC_10_ of glycine (15 μM) in presence and absence of 100 mM ethanol (by co-application). **(B)** The graph summarizes the effect of ethanol concentrations (0–100 mM) on α1 (black squares) and α1β (open squares) GlyRs. Data show positive ethanol modulation in both conformations, being significantly attenuated in α1β GlyRs (*n* = 16 α1 and *n* = 10 α1β) [*P* = 0.0014, *F*(4,28) = 5.941]. **(C)** Representative evoked current traces recorded in homomeric α2 and heteromeric α2β GlyRs activated with an EC_10_ of glycine (20 μM) with and without ethanol. **(D)** The graph summarizes the effect of ethanol concentrations on α2 (blue circles) and α2β (open blue circles) GlyRs. Data show positive ethanol modulation only in α2β GlyRs, being significant when compared with α2 (*n* = 12 α2, *n* = 15 α2β). Data are mean ± SEM. **p < 0.01, ***p < 0.001. Two-way ANOVA and Sidak’s multiple comparisons test.

Next, we tested the sensitivity of the different homomeric and heteromeric receptor conformations to ethanol using the EC_10__–__20_ determined from the data in Table 1. The low concentration of the agonist used in this experiment is related to its property of acting as a positive allosteric modulator (PAM), where its largest effect is at EC_10__–__20_ (Aguayo et al., 1996). First, we tested the sensitivity of α1 homomeric GlyRs to increasing concentrations of ethanol and found that 50 and 100 mM potentiated the peak current activated with 15 μM glycine (Figures 2A,B, closed squares). The data also showed that the potentiation of glycine-mediated currents was smaller in α1β heteromeric GlyRs, mostly at higher ethanol concentrations (50 and 100 mM). For example, at 100 mM of ethanol, the potentiation was 46 ± 4% in α1 and 25 ± 5% in the α1β conformer (*p* < 0.001, Figures 2A,B and Table 1). In agreement with a previous study (Yevenes et al., 2010), α2 homomeric GlyRs activated with 20 μM glycine were insensitive to 100 mM ethanol (12 ± 4%, Figures 2C,D, closed circles). However, α2β heteromeric GlyRs showed a higher sensitivity at concentrations as low as 10 mM (31 ± 7% of control, *p* = 0.035, Two-way ANOVA, Sidak’s multiple comparisons test), 50 mM (65 ± 9% of control, *p* < 0.0001, Two-way ANOVA, Sidak’s multiple comparisons test) and up to 100 mM ethanol (90 ± 20% of control, *p* = 0.0056, Two-way ANOVA, Sidak’s multiple comparisons test, Figures 2C,D and Table 1). Thus, the more significant effect of co-expressing β with α subunits is the increase in sensitivity to ethanol with the α2 containing subunit [ethanol × GlyR subunit composition interaction: *p* = 0.0001, *F*(4,90) = 7.271, Two-way ANOVA].

### Effect of the β Subunit on the Action of Ethanol at the Single Channel Level

**FIGURE 3:**
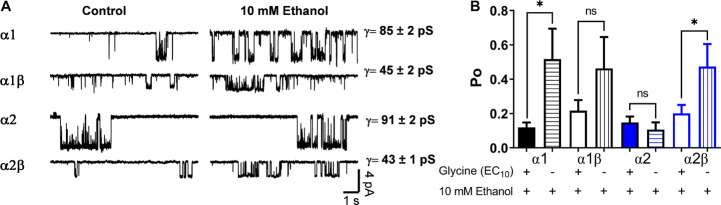
Effects of ethanol on single channels in homomeric and heteromeric GlyRs. **(A)** The traces are single channel recordings obtained in transfected HEK 293 cells with different GlyRs conformations before and after the application of 10 mM EtOH in presence of EC_10_ glycine. **(B)** The bar graph shows the percentage of change of open probability (Po) after the application of 10 mM EtOH in homomeric and heteromeric α1- and α2 GlyRs. The values show a decrease in heteromeric α1β GlyRs (*p* = 0.0211, t14 = 2.598, *n* = 8 α1, and *n* = 8 α1β) and an increase in the open probability of α2β GlyRs (*p* < 0.0001, t13 = 5.98, *n* = 8 α2, and *n* = 7 α2β). The single channel conductance was not affected by ethanol. Data are mean ± SEM, ^∗^*p* < 0.05, ^∗∗∗^*p* < 0.001. Unpaired Student’s *t*-test. ns, non significant.

To further characterize the effects of a low ethanol concentration (10 mM) on the homomeric and heteromeric GlyRs conformations, we recorded single channel activity using the outside out configuration with glycine alone as control (10 μM for α1, 20 μM for α2, and 10 μM for α2β) and comparing with 10 mM ethanol in the same recording. In α1 homomeric GlyRs, ethanol altered the Po and frequency of opening (Figures 3A,B and Table 2). For example, open probability in α1 homomeric GlyRs increased by 300% above control (control: 0.12 ± 0.03 vs. 10 mM: 0.52 ± 0.18; *p* < 0.001, paired *t*-test, Figures 3A,B and Table 2). The heteromeric α1β conformation showed a smaller increase in open probability than homomeric α1 subunits after ethanol application (control: 0.22 ± 0.06 vs. 10 mM: 0.47 ± 0.18; Unpaired *t*-test, Figure 3B and Table 2). In agreement with the whole-cell results, α2 homomeric GlyRs showed no changes in this parameter with 10 mM ethanol (control: 0.15 ± 0.03 vs. 10 mM: 0.11 ± 0.04; Figure 3B and Table 2). On the other hand, heteromeric α2β GlyRs were markedly affected by ethanol as reflected by an increase in open probability (control: 0.17 ± 0.03 vs. 10 mM: 0.45 ± 0.1; *p* < 0.001, paired *t*-test, Figures 3A,B and Table 2). The data shows that β subunit co-expression with α decreased channel conductance between homo-and heteromeric GlyRs (85–90 vs. 45 pS, respectively), and that no differences were found in the values of channel conductance in the presence of ethanol in any of the different subunit conformations (Figure 3A and Table 2).

**TABLE 2:** Effects of ethanol on the single channel properties for different conformations.

GlyR	Control				10 mM ethanol			
	Po	Conductance (pS)	Frequency (Hz)	*n*	Po	Conductance (pS)	Frequency (Hz)	*n*
α1	0.12 ± 0.03	85 ± 2	37 ± 9	8	0.52 ± 0.18 (***)	85 ± 2	55 ± 9 (*)	8
α1β	0.22 ± 0.06	45 ± 2	56 ± 8	8	0.47 ± 0.18	47 ± 1	64 ± 10 (**)	8
α2	0.15 ± 0.03	91 ± 2	37 ± 11	8	0.11 ± 0.04	89 ± 1	44 ± 14	8
α2β	0.17 ± 0.03	43 ± 1	58 ± 14	7	0.45 ± 0.1 (***)	44 ± 1	79 ± 11 (*)	7
α2AAβ	0.16 ± 0.06	45 ± 1	44 ± 3	10	0.14 ± 0.06	45 ± 1	43 ± 3	10

Values are given as mean ± SEM. The EC_10_ calculated for all subunits was used for out side out ethanol sensitivity recordings (−60 mV). Absolute values were statistically analyzed using the paired t-test and *p < 0.05 was statistically significant comparing with 10 mM Ethanol. n = (number of neurons).

**FIGURE 4:**
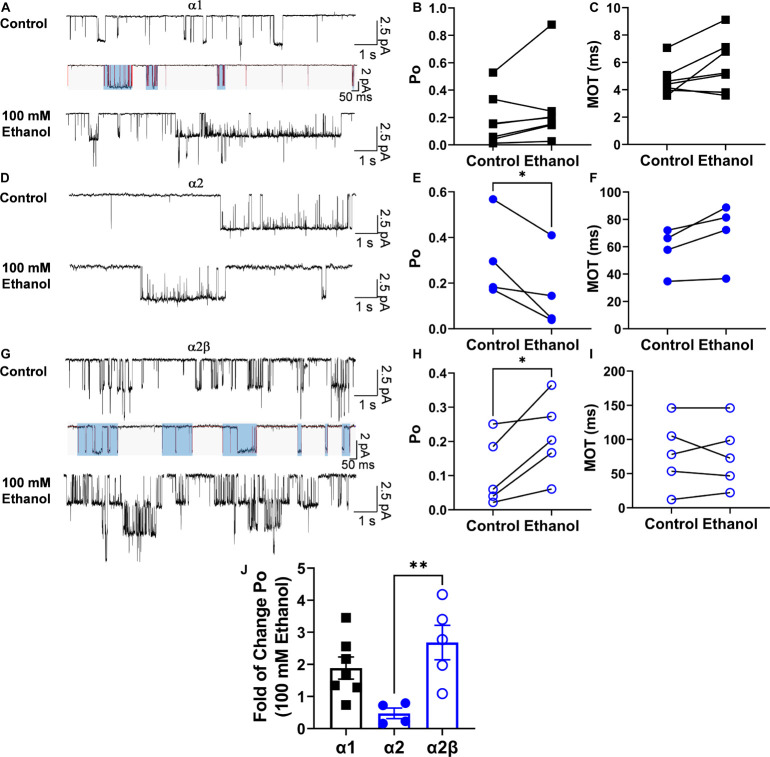
Positive effect of ethanol on Po in the α2β conformation. **(A)** Single channel representative traces for homomeric α1 GlyRs in the absence and presence of 100 mM ethanol. The lower traces depict burst analysis of single-channel records. Lines (*red*) indicate idealized traces, and boxes (*light blue*) indicate bursts, which are considered to represent individual activations of the receptor. Bursts for each subunit composition were determined on the basis of a critical shut time (*see* section “Materials and Methods”). **(B,C)** The graphs illustrate that the values for Po and mean open time (MOT) show a tendency to increase with 100 mM ethanol. **(D)** Single channel representative traces for homomeric α2 GlyRs in the absence and presence of 100 mM ethanol. **(E,F)** The value for Po was significantly decreased by 100 mM ethanol without changes in MOT. **(G)** Single channel representative traces for homomeric α2β GlyRs in the absence and presence of 100 mM ethanol. Lines (red) indicate idealized traces, and boxes (light blue) indicate bursts, which are considered to represent individual activations of the receptor. Bursts for each subunit composition were determined on the basis of a critical shut time (see section “Materials and Methods”). **(H,I)** The bars show that Po was significantly increased by 100 mM ethanol without changes in MOT. **(J)** The bar graph shows an increase in the fold of change of the Po during the application of 100 mM ethanol in α1 and α2β GlyRs showing a significant difference between α2 and α2β GlyRs. Data are mean ± SEM, *p < 0.05, **p < 0.01. Paired Student’s *t*-test **(A–I)** and Unpaired Student’s *t*-test **(J).**

A previous study proposed a kinetic model to explain the effects of ethanol in the α1 subunit (Welsh et al., 2009). Therefore, using a similar concentration of ethanol to that tested previously (Welsh et al., 2009), the effects of ethanol (100 mM) on the α2β conformation were analyzed (Figure 4). In homomeric α1 GlyRs, the results showed that this concentration of ethanol did not affect open probability (Po, *p* = 0.1619, t9 = 1.6, Paired *t*-test, *n* = 7), frequency of opening (5,483 ± 2,323 ctrl events vs. 7,166 ± 3,412 ethanol events, *p* = 0.3428, t6 = 1.03, Paired *t*-test) and mean open time (4.7 ± 0.4 ms ctrl vs. 5.8 ± 0.7 ms ethanol, *p* = 0.07, t6 = 2.198, Paired *t*-test) (Figures 4A–C). On the other hand, in α2 homomeric GlyRs, ethanol decreased Po (0.3 ± 0.09 ctrl vs. 0.16 ± 0.08 ethanol, *p* = 0.045, t3 = 3.322, Paired *t*-test) and the frequency of opening (928.3 ± 660.8 ctrl events vs. 705.3 ± 620.2 ethanol events, *p* = 0.02, t3 = 4.401, Paired *t*-test) (Figures 4D–F). In α2β receptors, however, ethanol enhanced Po (0.11 ± 0.05 ctrl vs. 0.21 ± 0.05 ethanol, *p* = 0.029, t4 = 3.336, Paired *t*-test) and with a tendency to increase the frequency of opening (2,746 ± 2,073 ctrl events vs. 4,782 ± 2,166 ethanol events, *p* = 0.087, t4 = 2.25, Paired *t*-test) (Figures 4G–I). Altogether, ethanol enhanced Po in both α1 (1.89 ± 0.35) and α2β, but not in α2 (α2: 0.47 ± 0.16 vs. α2β: 2.68 ± 0.54 ethanol, *p* = 0.0097, t7 = 3.519, unpaired *t*-test) (Figure 4J). However, mean open time was not modified by ethanol (Figure 4H). In addition, analysis of intraburst open probabilities showed no differences in the α1 and α2β subunit combinations (0.70 ± 0.040 vs. 0.64 ± 0.041, respectively; *P* > 0.05, two-tailed *T* test). In α1 subunit glycine receptors, ethanol increased burst duration (28 ± 5.2 vs. 42 ± 2.2 ms for control and ethanol, respectively; *P* < 0.01, paired *t*-test) without altering intraburst Po (0.70 ± 0.040 vs. 0.57 ± 0.052 for control and ethanol, respectively; *P* > 0.05, paired *t*-test). In α2β subunit glycine receptors, ethanol did not alter burst duration (89 ± 15 vs. 66 ± 12 ms for control and ethanol, respectively; *P* > 0.05, paired *t*-test) or intraburst Po (0.64 ± 0.041 vs. 0.69 ± 0.019 for control and ethanol, respectively; *P* > 0.05, paired *t*-test). Although the addition of the β subunit to the α2 GlyR conferred ethanol sensitivity similar to that seen in α1 GlyRs, the kinetics of the α2β GlyR nevertheless differed from those of the α1 GlyR. For example, while open probability within individual receptor activations (bursts) was similar, burst duration and intraburst mean open and shut times differed considerably between α1 and α2β GlyRs (Table 3).

**TABLE 3:** GlyR Burst Analysis.

	Intraburst open times				Intraburst shut times				
GlyR	τ_1_ (ms) (Area)	τ_2_ (ms) (Area)	τ_3_ (ms) (Area)	Mean (ms)	τ_1_ (ms) (Area)	τ_2_ (ms) (Area)	Mean (ms)	Mean burst length (ms)	Intraburst P_o_
α_1_	0.48 ± 0.076 (35 ± 2.3)	2.7 ± 0.35 (46 ± 3.2)	15 ± 2.0 (20 ± 2.6)	4.6 ± 0.58	0.50 ± 0.045 (77 ± 3.6)	4.2 ± 2.1 (9.7 ± 1.8)	2.5 ± 0.27	27.5 ± 5.24	0.70 ± 0.040
α2β	0.31 ± 0.12 (26 ± 5.7)	4.4 ± 2.1 (31 ± 10)	20 ± 1.7 (51 ± 12)	13 ± 2.3**	0.20 ± 0.0020 (27 ± 4.2)	2.6 ± 0.63 (54 ± 9.9)	9.3 ± 2.6*	88.8 ± 14.6**	0.64 ± 0.041

*P < 0.05, **P < 0.01; two-tailed T-test. n = 6 and 4 cells for α1 and α2β, respectivelly.

### The Effects of Ethanol on α2β Glycine Receptors Is Mediated by a Gβγ-Linked Mechanism

**FIGURE 5:**
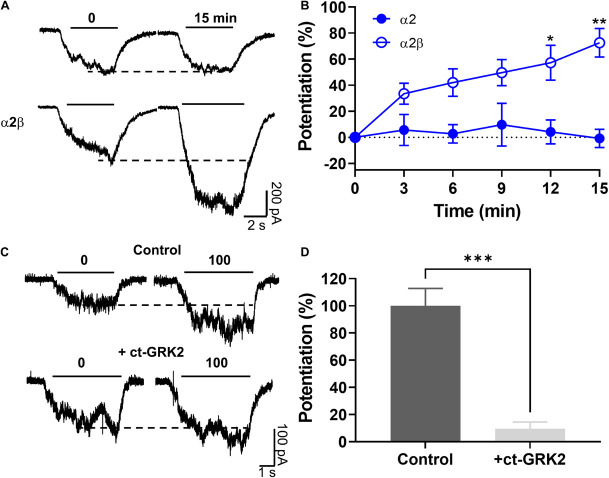
The ethanol induced potentiation in α2β GlyRs was mediated by a Gβγ-linked mechanism. **(A,B)** Representative evoked current traces from α2 and α2β conformations showing the effects of G-protein activation by intracellular dialysis of GTP-γ-S (0.2 mM) for 15 min. The time course graph summarizes the effects of G-protein activation by GTP-γ-S in α2 (closed blue circles) and α2β (open blue circles). A significant potentiation was found only in α2β GlyRs. **(C)** Current traces from HEK 293 cells transfected with α2β GlyRs, with or without the co-expression of the Gβγ scavenger ct-GRK2. **(D)** The graph summarizes the percentage potentiation elicited by 100 mM ethanol on α2β GlyRs in the absence or presence of ct-GRK2 in an independent experiment. Data are mean ± SEM. *p < 0.05, **p < 0.01, and ***p < 0.001. Two-way ANOVA and SIdak’s multiple comparisons test **(B)** and Unpaired Student’s *t*-test **(D)**.

Previous reports using neuronal and recombinant α1 GlyRs showed that the amplitude of the glycine-activated current was strongly enhanced after 15 min of intracellular dialysis with GTP-γ-S, implying that Gβγ enhances GlyRs activity (Yevenes et al., 2003, 2010). To investigate the dependency of G protein activation on the potentiation of α2β GlyRs by ethanol, we transfected α2 in different subunit conformations in HEK 293 cells. The data showed that after 15 min of intracellular dialysis, a large current enhancement was found in α2β heteromeric GlyRs (73 ± 11%), but not in α2 homomeric GlyRs (−1 ± 7%, *p* = 0.0013, *F*(3,15) = 5.03, Two-way ANOVA, Sidak’s multiple comparisons test; Figures 5A,B and Table 1). Therefore, the data indicates that the β subunit confers the properties for G protein modulation. Thus, these data demonstrate the importance of Gβγ signaling for the ethanol effects on α2 GlyRs. Employing a widely used approach to examine the involvement of Gβγ, we expressed a Gβγ scavenger protein, ct-GRK2, that binds with high affinity to this dimer (Yevenes et al., 2003, 2008). Cells transfected with ct-GRK2 should not be potentiated by ethanol because ct-GRK2 binds “free” Gβγ and prevents its interaction with effectors. Overexpression of ct-GRK2 in an independent experiment show a significant attenuation of the potentiation induced by 100 mM ethanol in α2β GlyRs (control: 100 ± 13% vs. ct-GRK2: 10 ± 5%; *p* < 0.001, t7 = 7.313, Unpaired *t*-test, Figures 5C,D) strongly indicating that the Gβγ signaling is critical for ethanol effects on α2β-containing GlyRs.

**FIGURE 6:**
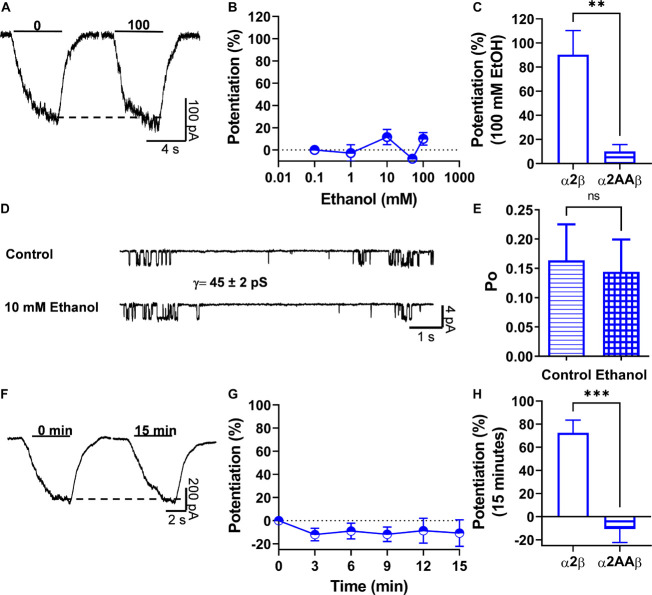
Two basic residues in the IL of α2 subunits are important for Gβγ-ethanol mediated effects. **(A)** Representative evoked current traces from heteromeric α2AAβ GlyRs showing the effects of 100 mM ethanol measured with an EC_10_ of glycine (20 μM). **(B)** The graph summarizes the effect of several ethanol concentrations on α2AAβ (half open blue circles) GlyRs. Data showed that ethanol did not potentiate α2AAβ GlyRs (*n* = 8). **(C)** Bar graph showing the potentiating effect of 100 mM ethanol on the peak amplitude of the glycine-activated current in heteromeric α2β GlyRs and the introduced mutation in α2 that makes the heteromeric GlyRs resistant to ethanol. **(D)** Single channel recordings from transfected HEK 293 cells with α2AAβ GlyRs before and after the application of 10 mM EtOH. **(E)** The bar graph shows the change of open probability (Po) after the application of 10 mM EtOH in heteromeric α2AAβ GlyRs. The values show an increase in Po in heteromeric α2β GlyRs. This increase was blocked with the AA mutation in the KR residues present in the IL. **(F,G)** Representative evoked current traces from α2AAβ showing the effects of G-protein activation by intracellular dialysis of GTP-γ-S (0.2 mM) for 15 min. The time course graph summarizes the effects of G-protein activation by GTP-γ-S in α2AAβ (half open blue circles). **(H)** Bar graph summarizes the effects of G-protein activation after 15 min showing the potentiation of the glycine evoked current in α2β GlyRs. Data are mean ± SEM. **p < 0.01, ***p < 0.001, ns, non significant. Unpaired Student’s *t*-test with Welch’s correction **(C)**, Unpaired Student’s *t*-test **(E,H)**.

It was previously reported that the α2 subunit has the molecular determinants in the intracellular domain necessary for allosteric modulation of GlyRs via activation of Gβγ (Yevenes et al., 2010). Here, two basic amino acids (K389 and R390) in the large intracellular loop of the α2 GlyRs subunit were detected and they were homologous to residues present in the α1 GlyRs subunit (K385 and K386) that are critical for binding and functional modulation by ethanol and Gβγ (Yevenes et al., 2006, 2008, 2010). In this study, we found that changing these two basic amino acids to alanine in α2 and co-expressing the mutant with the β subunit decreased the EC_50_ value compared to α2 homomeric GlyRs (*p* < 0.001, Figure 1E and Table 1). More interesting, the mutation abolished the ethanol-induced potentiation present in α2β heteromeric GlyRs (90 ± 20% vs. 10 ± 6%, *p* = 0.001, t20 = 3.86, Unpaired *t*-test with Welch’s correction) (Figures 6A–C and Table 1). Single channel recordings confirmed that the mutation of these two residues in α2 conferred resistance against low ethanol concentration effects (Po control: 0.16 ± 0.06 vs. Po 10 mM: 0.14 ± 0.06; Figures 6D,E and Table 2), being significantly reduced compared with the naïve α2 subunit (143 ± 27% vs. 6.6 ± 33%, *p* = 0.098, t15 = 2.957, Unpaired *t*-test, Figure 6E). Mechanistically, the activation of G-protein after 15 min of intracellular dialysis with GTP-γ-S showed that the α2AA mutation in the intracellular loop conferred resistance to potentiation by Gβγ when co-transfected with the β subunit (73 ± 11% vs. −11 ± 11%, *p* < 0.001, t18 = 5.239, Unpaired *t*-test; Figures 6F–H and Table 1). Thus, these results support the idea that these two residues and the β subunit are important for α2 GlyR modulation by ethanol.

### Beta Subunits Affect the Pharmacology of α2 Glycine Receptors to Positive Allosteric Modulators

**FIGURE 7:**
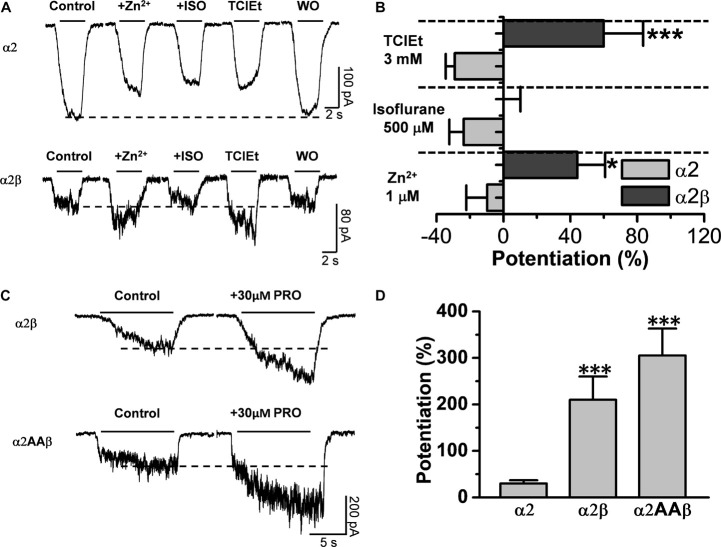
The β subunit confers α1-like pharmacological properties to α2β GlyRs regarding several positive allosteric modulators (PAMs). **(A)** Representative traces of glycine evoked currents in the presence of Zinc (1 μM), isoflurane (500 μM), and trichloroethanol (3 mM) on HEK 293 cells expressing α2 and α2β GlyRs. **(B)** The bar graphs show the effects of co-expressing the β subunit on the sensitivity to Zinc and TClEt. No significant effect was found with isoflurane. **(C)** Glycine evoked current traces in transfected HEK 293 cells with α2β and α2AAβ GlyRs before and after 30 μM propofol application. **(D)** The β subunit confers sensitivity to propofol in α2β and α2AAβ expressing GlyRs. Data are mean ± SEM. *p < 0.05, ***p < 0.001. Unpaired Student’s *t*-test.

Several studies have reported different molecular sites in transmembrane 2 and 3 (TM2 and TM3) within the α1 subunit that are important for the actions of ethanol and other allosteric modulators (Mascia et al., 1996, 2000; Mihic et al., 1997; Lobo et al., 2004; Borghese et al., 2012). Furthermore, several PAMs differentially affect homomeric α1 and α2 GlyRs (Yevenes and Zeilhofer, 2011b). Therefore, we wanted to characterize whether the incorporation of the β subunit affected the modulation to some typical PAMs in α2 GlyRs. The data show that α2 homomeric GlyRs are inhibited by the applications of Zn^2+^, isoflurane, and trichloroethanol (Figures 7A,B). The data also show that the β subunit causes a reversal from inhibition to potentiation of the α2β complexes in presence of trichloroethanol (α2: −29 ± 5% vs. α2β: 60 ± 24%; *p* = 0.0005, t21 = 4.152, *n* = 13 α2, and *n* = 10 α2β, Figures 7A,B) and Zn^2+^ (α2: −10 ± 13% vs. α2β: 44 ± 17%; *p* = 0.0151, t21 = 2.647, *n* = 13 α2, and *n* = 10 α2β, Figures 7A,B). The finding that isoflurane was unable to produce a potentiating action in α2 and α2β supports the notion that α2 containing GlyRs are not molecular targets for this PAM (α2: −24 ± 9% vs. α2β: 0 ± 10%; *p* = 0.0879, t20 = 1.794, *n* = 13 α2, and *n* = 9 α2β, Figures 7A,B). Another classical PAM is propofol, which has been shown to potentiate α1-mediated GlyRs currents (Moraga-Cid et al., 2011) and to modulate glycinergic synaptic transmission in medium spiny neurons (MSNs) in the nAc (Munoz et al., 2018). In addition, a single phenylalanine residue (F380 in IL) was found to be critical on this effect in α1 GlyRs (Moraga-Cid et al., 2011). Our data show that α2 homomeric GlyRs were potentiated to a small extent by propofol (30 ± 7% of control) (Figure 7D and Table 1), whereas both α2β naive and its mutant version (α2AA) were significantly potentiated by the modulator as compared to α2 GlyRs (α2β: 210 ± 50%, α2AAβ: 306 ± 58%; α2 vs. α2β, *p* < 0.0001, t11 = 9.613, *n* = 6 α2 and *n* = 7 α2β, α2 vs. α2AAβ, *p* = 0.0009, t12 = 4.094, *n* = 8 α2AAβ, Figures 7C,D and Table 1). Thus, the data suggest that the β subunit can exert critical sensitivity to several PAMs in an α2 expressing channel.

## Discussion

### A New Modulatory Role for β Subunits in the Function of Glycine Receptors

The present study provides new information about a previously unrecognized role of the β subunit in the allosteric modulation of GlyRs by an important group of depressants, i.e., ethanol and general anesthetics. Additionally, it provides evidence for the critical role of basic residues present in the IL of the α2 subunit on its functional modulation by Gβγ and ethanol. Furthermore, the present data support the conclusion that GlyRs expressing α2β are one of the most sensitive brain targets for ethanol allosteric modulation.

Classically, the β subunit of GlyRs has been widely understood to act as a structural component in the receptor because it does not present the molecular requirement for glycine binding and Cl^–^ ion permeation (Grudzinska et al., 2005; Weltzien et al., 2012). The β subunit contributes to GlyR physiology reducing single channel conductance (Table 2), affects the pharmacology of the GlyR complex (Table 1), and has a key role in the generation of startle disease (James et al., 2013; Piro et al., 2021). Together with α subunits, the β subunit forms heteropentameric receptors having all the properties of native receptors, i.e., highly selective to its natural agonist, inhibited by strychnine, and modulated by several PAMs such as Zn^2+^, propofol, and ethanol (Yevenes and Zeilhofer, 2011a). Also, together with a peripheral protein (gephyrin), the β subunit has a receptor anchoring function that localizes the GlyRs to the postsynaptic region (Grudzinska et al., 2005; Zeilhofer et al., 2005; Weltzien et al., 2012). Heteromeric GlyRs, however, have also been found at extrasynaptic locations where they mediate tonic glycinergic inhibition in the spinal dorsal horn (Gradwell et al., 2017), supporting a structural and anchoring role in sensorial pathways. Because α2β heteromeric conformations are found in the central nervous system (Forstera et al., 2017), it is likely that they contribute to the effect of ethanol on the tonic current induced by glycine in the nucleus accumbens (Munoz et al., 2020). However, the homomeric α2 GlyR was found to be insensitive to ethanol (Sanchez et al., 2015).

The present study provides functional evidence supporting a modulatory role for α2 containing GlyRs. For example, we found that co-expression of the β subunit in heteromeric GlyRs makes the α2β configuration more sensitive to glycine (Table 1), ethanol, and to several pharmacologically relevant PAMs. Furthermore, mutation of two amino acids in the IL that were suggested to be related to Gβγ binding and modulation of α1 subunits (Yevenes et al., 2010) abolished the potentiation of the α2β GlyRs by ethanol and GTP-γ-S. Additionally, co-expression of ct-GRK2, a ligand with Gβγ blocking properties (Yevenes et al., 2003), significantly reduced the potentiation by ethanol in α2β GlyRs adding additional support to the notion that the β subunit changes the functional properties of α2-containing GlyRs. Thus, the α2β conformer shows an α1-like pharmacological phenotype and adds new information about the molecular requirements for several clinically relevant PAMs (Mascia et al., 1996, 2000; Mihic et al., 1997; Yevenes and Zeilhofer, 2011a; McCracken et al., 2013).

### Implication for the Presence of β Containing Glycine Receptors in the Upper Brain

Although GlyRs have been routinely linked to neuronal inhibition of spinal regions (Legendre, 2001; Lynch, 2004; Zeilhofer et al., 2012), more recent studies have reported the expression of α2 and α3 subunits in supra spinal regions (Salling and Harrison, 2014; Forstera et al., 2017; McCracken et al., 2017; Munoz et al., 2018, 2020; Gallegos et al., 2019) being primarily related to ethanol (Forstera et al., 2017; McCracken et al., 2017; Gallegos et al., 2019; Munoz et al., 2020) and propofol actions (Munoz et al., 2018). Using KO mice, it was suggested that α2- and α3-containing GlyRs are important for sustaining tonic currents in the forebrain (McCracken et al., 2017) and that they might contribute to ethanol consumption (Blednov et al., 2015). These published results are interesting because while homomeric α2 or α3 GlyRs are insensitive to ethanol (Yevenes et al., 2010; Sanchez et al., 2015), any effect on animal behavior should be associated to the expression of β subunits in α2-containing GlyRs, or to some receptor compensation in the KO mice. Recent studies provided experimental evidence supporting the expression of α1, α2, and α3 subunits in the nAc in WT mice (Forstera et al., 2017). In addition, using KI mice with the same mutation in α2 (K389 and R390), we demonstrated the presence of α2β GlyRs in accumbal neurons (Gallegos et al., 2021). Interestingly, α2 KI mice showed reduced sedation and increased ethanol consumption, suggesting that the α2 subunit is important for the ethanol potentiation of GlyRs in the adult brain (Gallegos et al., 2021). The insensitivity to ethanol of glycinergic synaptic currents in the nAc suggests that these GlyRs are mainly composed of α1β conformations (Munoz et al., 2018). Therefore, the broad expression of α and β subunits likely play a role in several behavioral conduits and might allow for the development of pharmacotherapy based on the presence of these ethanol sensitive targets.

### Potential Mechanisms for the Conversion of the α2 Subunit Into an “α1-Like” Heteromeric Complex

**FIGURE 8:**
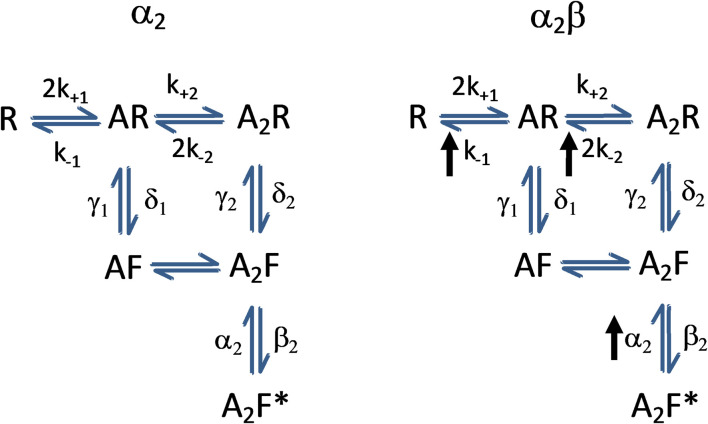
Scheme legend. GlyR kinetic model from Krashia et al. (2011) showing possible changes in GlyR α2 kinetics (left) due to the expression of the β subunit (right) and that could account for the observed differences in ethanol modulation. The proposed increase in closing rate α is based on the observations of decreased mean open time in α2β vs. α2 homomeric subunits, whereas the increases in glycine dissociation rates are proposed based on both the reported action of ethanol to decreased glycine unbinding rate (Welsh et al., 2009) and the lower glycine potency observed in the present study. R, receptor; A, agonist; F, “flipped” (pre-activated) state. Note that the proposed changes to the model are hypothetical and further analysis should test it in detail.

In an attempt to understand how the addition of the β subunit to the α2 subunit was able to confer sensitivity to several PAMs, and primarily to ethanol, we can consider a previously published model for GlyRs gating (Krashia et al., 2011). The study on the α1 subunit showed that ethanol did not affect channel conductance or open and closed dwell times and likelihoods of the channel. On the other hand, the main effect of ethanol on α1 function was the enhancement of burst durations (Welsh et al., 2009) which likely reflected a decreased glycine unbinding rate(s) (k-1 and/or 2k-2), without affecting other transitions (Kirson et al., 2017), which resulted in a prolongation of burst durations without a change in mean open time (Welsh et al., 2009). The present results show that ethanol increased open probability (Po) and frequency of opening without affecting mean open time in α1 GlyRs, but decreased Po and frequency of opening in α2 GlyRs. In α2β GlyRs, ethanol enhanced both Po and frequency of opening in a manner similar to that found in the α1 subunit. On the other hand, we found that ethanol did not change the intraburst MOT or intraburst Po or burst duration in the α2β. In addition, expression of the β subunit in α2 receptors shortened mean open time to approximately that of the α1 subunit, indicating an increase in closing rate. The higher affinity of α2 receptors for glycine compared to α1 receptors, which results from multiple kinetic rates rather than a simple change in unbinding rate (Lape et al., 2008; Krashia et al., 2011), may render this conformer resistant to the potentiating effects of ethanol in the absence of the β subunit (see scheme, Figure 8).

Our results suggest that the β subunit might rearrange the states of GlyRs changing the kinetics at the single channel level. It is possible that the β subunit interspaced with α2 allows the exposure of key residues important for the conformational changes occurring after agonist binding (Lape et al., 2008; Krashia et al., 2011). They might be complementary to those recently reported that showed that shortening the IL in human GlyRs increased the open probability. The model proposed was that the IL has a modulatory action on GlyRs gating by introducing tension between TM3 and TM4 and causing them to reorient during channel opening (Ivica et al., 2020).

## Conclusion

We describe a new role for the β subunit for modulation by ethanol and other PAMs in α2 containing GlyRs. Additionally, our study supports the notion that heteromeric β expressing GlyRs might play a crucial role in the control of excitability in upper brain regions that express α2 subunits.

## Data Availability Statement

The raw data supporting the conclusions of this article will be made available by the authors, without undue reservation.

## Author Contributions

BM and LA designed the study and wrote the manuscript. BM performed and analyzed most whole cell experiments. RP performed and analyzed the single channel studies. TM, PM, CP, and GM-C assisted in the electrophysiological experiments. BM, GY, GM-C, and LA corrected and discussed the manuscript. LA obtained the funding to support the study, guided and discussed the experiments, and corrected the manuscript. All authors revised and approved the final version of the manuscript.

## Conflict of Interest

The authors declare that the research was conducted in the absence of any commercial or financial relationships that could be construed as a potential conflict of interest.

## Publisher’s Note

All claims expressed in this article are solely those of the authors and do not necessarily represent those of their affiliated organizations, or those of the publisher, the editors and the reviewers. Any product that may be evaluated in this article, or claim that may be made by its manufacturer, is not guaranteed or endorsed by the publisher.
